# Cancer Stem Cells and Macrophages: Implications in Tumor Biology and Therapeutic Strategies

**DOI:** 10.1155/2016/9012369

**Published:** 2016-02-14

**Authors:** Bruno Sainz, Emily Carron, Mireia Vallespinós, Heather L. Machado

**Affiliations:** ^1^Department of Biochemistry, Autónoma University of Madrid, School of Medicine, 28018 Madrid, Spain; ^2^Instituto de Investigaciones Biomédicas “Alberto Sols”, CSIC and UAM, 28018 Madrid, Spain; ^3^Department of Biochemistry and Molecular Biology, Tulane Cancer Center, Tulane School of Medicine, New Orleans, LA 70118, USA

## Abstract

Cancer stem cells (CSCs) are a unique subset of cells within tumors with stemlike properties that have been proposed to be key drivers of tumor initiation and progression. CSCs are functionally defined by their unlimited self-renewal capacity and their ability to initiate tumor formation* in vivo*. Like normal stem cells, CSCs exist in a cellular niche comprised of numerous cell types including tumor-associated macrophages (TAMs) which provides a unique microenvironment to protect and promote CSC functions. TAMs provide pivotal signals to promote CSC survival, self-renewal, maintenance, and migratory ability, and in turn, CSCs deliver tumor-promoting cues to TAMs that further enhance tumorigenesis. Studies in the last decade have aimed to understand the molecular mediators of CSCs and TAMs, and recent advances have begun to elucidate the complex cross talk that occurs between these two cell types. In this review, we discuss the molecular interactions that define CSC-TAM cross talk at each stage of tumor progression and examine the clinical implications of targeting these interactions.

## 1. Introduction

**Figure 1:**
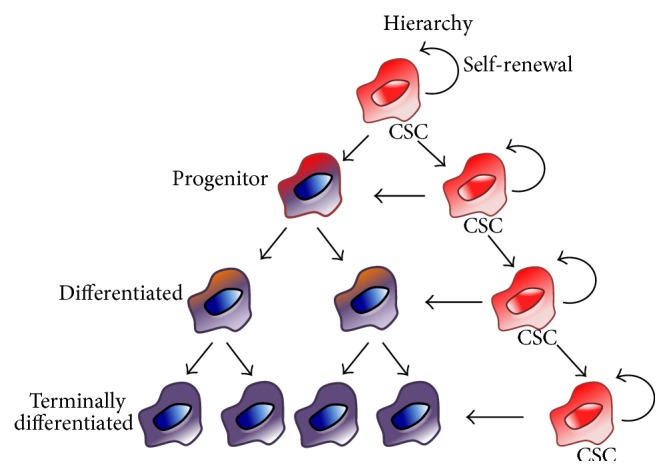
The CSC model. Over time, CSCs acquire phenotypes and characteristics of normal stem cells such as unlimited self-renewal and the capacity to divide indefinitely and at the same time maintain the ability to generate multiple cell lineages, including differentiated progenies. A CSC can thus divide (1) asymmetrically (differentiation) giving rise to one CSC and a specialized differentiated cell or (2) symmetrically (self-renewal) giving rise to two identical CSCs. In both cases, the capacity of self-renewal remains intact and ensures the survival of the CSC pool and supports the hierarchical model of tumor cell heterogeneity.

Cancer stem cells (CSCs), also known as tumor-initiating cells or tumor-propagating cells, constitute a biologically unique subset of stemlike cells within the bulk tumor cell population. These cells are hypothesized to be key drivers of the multistep process of oncogenesis, giving rise to the clonogenic core of tumor tissues. Thus, according to the CSC model of tumor heterogeneity [1], malignancies have a hierarchical developmental structure with the CSC at the top of the hierarchy (Figure 1). This idea that tumor initiation and progression are driven by stemlike cells was first proposed >150 years ago by Virchow [2] and has long been debated. While their existence has been confirmed across numerous different tumor entities, including acute myeloid leukaemia [3], pancreatic cancer [4, 5], breast cancer [6], lung cancer [7], hepatocellular carcinoma [8], head and neck cancer [9], colon cancer [10, 11], prostate cancer [12], melanoma [13, 14], and glioblastoma [15], the origin of CSCs is not fully understood. This review does not aim to discuss the origin of CSCs, except to point out that whether CSCs arise from normal stem/progenitor/differentiated cells or acquire mutations that confer stem cell-like properties, CSCs should not be confused with normal stem cells becoming cancerous (“cancerous stem cells”) [16]. Rather CSCs are believed to have acquired, over time, phenotypes and characteristics of normal stem cells such as unlimited self-renewal and the capacity to divide indefinitely and at the same time maintain the ability to generate multiple cell lineages, including differentiated progenies [17, 18]. Thus, CSCs are functionally defined by their self-renewal capacity, their multipotency, and their exclusive ability to initiate tumors in mice upon serial passage [1, 16].

The clinical implication of the CSC model suggests that only elimination of the CSC will result in eradication of the tumor, while failure to do so will inevitably lead to tumor relapse. This concept is supported by data demonstrating that primary tumors with a clear stem cell signature are consistently associated with poor response rates and relapse [19–22], and CSCs are more resistant to chemotherapy and radiotherapy than “differentiated” tumor cells [22, 23], likely due to cellular defense mechanisms shared with normal stem cells [24–26]. Consequently, the idea of eliminating CSCs as a therapeutic strategy is already beginning to revolutionize how we foresee cancer treatment in the immediate future, with CSC-specific compounds expected to lead the battle. However, we are far from achieving this goal, as our understanding of the CSC niche and the cellular determinants that CSCs need for survival is in its infancy.

**Figure 2:**
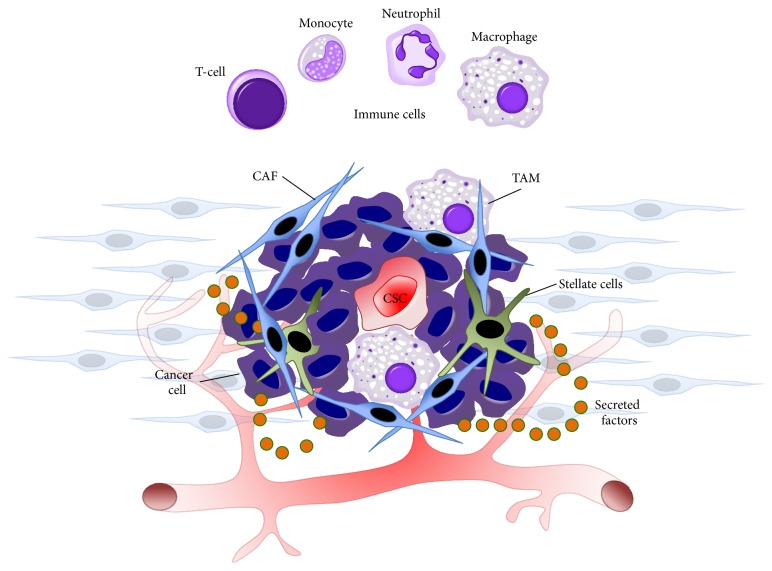
The CSC niche and tumor microenvironment. At center stage is the CSC, in contact with a complex and dynamic cellular network, including daughter cancer cells, stellate cells (in the case of HCC and PDAC), cancer-associated fibroblasts (CAFs), and immune cells, which include T-cells, monocytes, neutrophils, and tumor-associated macrophages (TAMs). Nourished by the circulatory system, these cells communicate with one another and directly with the CSC via secreted factors, forming a positive feedback loop that promotes CSC tumorigenicity and metastasis.

Like somatic stem cells, CSCs exist in a cellular niche that provides key signals for self-renewal and tumorigenesis [27, 28] (Figure 2). More specifically, the tumor microenvironment protects CSCs from immune surveillance, apoptosis, and chemotherapeutics and above all, the niche provides CSCs with factors that maintain, drive, and promote their “stemness.” In general, developing tumors promote the creation of a unique cellular microenvironment containing extracellular matrix proteins (e.g., collagen, elastin) and a diverse collection of cells, including cancer-associated fibroblasts; stellate cells [in pancreatic cancer or hepatocellular carcinoma (HCC)]; immune cells such as myeloid-derived suppressor cells, monocytes, macrophages, and T-cells; and endothelial cells [29–31]. While each cell or environmental component has a particular function on its own, together they create a dynamic niche replete with secreted factors that synergize and cooperate to develop a complex communication network known as cross talk, with the CSC at center stage.

The importance of the tumor microenvironment in promoting cancer initiation and tumor growth has been increasingly recognized over the past decade [31–35]. In addition to providing structural support for tumor development, the tumor-associated microenvironment of many solid tumors provides cues to CSCs that regulate their self-renewal and metastatic potential as well as their resistance to conventional chemotherapeutic agents [33, 36]. For example, in human breast cancers, recruited mesenchymal stem cells (MSC) interact with breast CSCs through cytokine loops involving interleukin- (IL-) 6, CXCL7, prostaglandin E2, IL-8, or Gro-*α* stimulating their self-renewal capacity [37, 38]. Stromal fibroblasts present in invasive human breast carcinomas promote tumor growth through elevated SDF-1/CXCL12 secretion [37], and lung stromal fibroblast-derived periostin creates a metastatic niche for breast CSCs [39]. In pancreatic cancer, tumor-associated pancreatic stellate cells create a paracrine niche for pancreatic CSCs via Nodal/Activin secretion [33]. Likewise, hepatic stellate cells in HCC contribute to liver CSC chemoresistance by secreting hepatocyte growth factor (HGF) [40]. These studies provide further evidence that the tumor microenvironment is essential for CSC functions.

An area of great interest is the role of inflammatory cells in the CSC niche. The tumor microenvironment is characterized by chronic inflammation, which, instead of inhibiting tumor growth, favors tumor formation by stimulating cell proliferation, activating CSCs, and promoting metastasis [28, 41]. Leading the tumor inflammatory response are tumor-associated macrophages (TAMs) [42]. A correlation between high numbers of TAMs and rapid disease progression and poor patient outcome has been observed for decades [32, 43, 44]; however, only recently was this paradoxical phenotype explained. We now understand that this correlation is due to TAM-mediated paracrine signaling, in which macrophage-derived factors activate the CSC compartment and promote stemlike features of CSCs, exacerbating tumor progression, metastasis, and even CSC chemoresistance. In this review, we focus on the role of TAMs in CSC biology and pathogenesis in solid tumors. We critically discuss the contribution of TAMs on premalignancy, primary tumor CSCs, circulating CSCs, and the initiation of premetastatic niches in distant organs. We also examine the prospects of directly targeting TAMs or disrupting TAM-CSC cross talk for cancer therapy.

## 2. Tumor-Associated Macrophages

**Figure 3:**
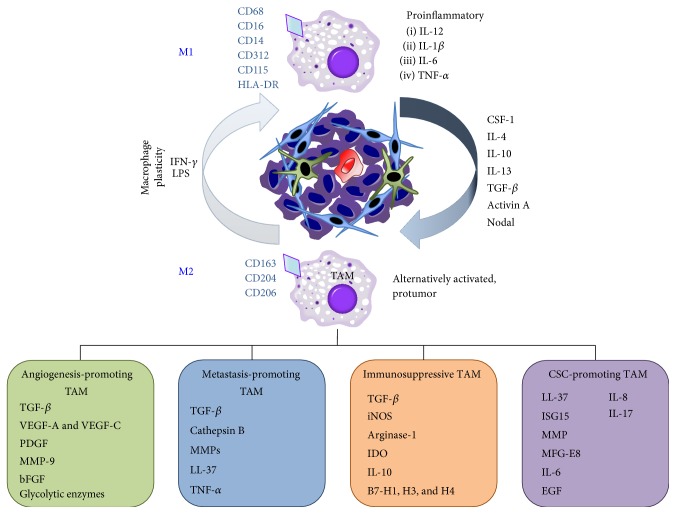
Macrophage plasticity and characterization. The binary M1/M2 classification of macrophages suggests that human macrophages exist as either proinflammatory M1 macrophages or protumor M2 TAMs, which can be identified based on the expression of cell surface cell membrane markers. This concept has been challenged by the identification of numerous TAM subtypes (angiogenesis-promoting TAM, metastasis-promoting TAM, immunosuppressive TAM, and CSC-promoting TAM) that exist within the primary tumor and metastatic sites. The existence of a specific TAM subtype is driven by the interaction of macrophages with factors secreted by the tumor microenvironment, leading to transcriptional rewiring of TAMs with a specific gene signature profile. TAMs are highly plastic and can shift between subtypes based on tumor-specific signals and stimuli.

Macrophages, a heterogeneous population of innate myeloid cells, originate from monocytic precursors and can undergo specific differentiation/polarization in the blood or within tissues [45, 46]. In addition to monocytes, the yolk sac and fetal liver represent two additional sources for colony-stimulating factor-1 receptor- (CSF-1R-) dependent macrophages during early development [47, 48]. Macrophages are not static but rather are extremely plastic and can assume multiple phenotypes in response to constantly changing environmental cues (e.g., bacterial infection, wounds, and cancer). From a simplistic point of view, macrophages are polarized towards a classically activated or “M1” phenotype via type I helper T (Th1) cytokines [e.g., interferon- (IFN-) *γ*] and/or activation of Toll-like receptors upon engagement with bacterial components (e.g., lipopolysaccharides). M1 macrophages are therefore involved in Th1 responses to pathogens and microbes and are characterized by elevated proinflammatory cytokines such as IL-12, IL-1*β*, IL-6, and tumor necrosis factor *α* (TNF-*α*), increased expression of major histocompatibility complex (MHC) class II, generation of reactive oxygen and nitrogen intermediates, and enhanced cell killing [49]. In response to IL-4, IL-10, and IL-13, however, macrophages can polarize towards an alternatively activated “M2” phenotype participating in Th2-type responses including humoral immunity, wound healing, and tissue remodeling [50]. They are characterized by high expression of scavenging molecules, mannose and galactose receptors, activation of the arginase pathway, production of IL-10, vascular endothelial growth factor (VEGF), and matrix metalloproteinases (MMPs), and efficient phagocytic activity [49, 50] (Figure 3).

Monocyte infiltration into a tumor is mediated by chemokines (e.g., CCL2, CCL5, and CXCL12), CSF-1, and components of the complement cascade [51, 52]. Once they are within the tumor, the tumor environment rapidly promotes their differentiation into tumor-conditioned macrophages. TAMs were initially believed to be biased away from an M1 phenotype, expressing M2 protumor markers [53]. We now understand that while they do share greater similarity with alternatively activated M2 macrophages, tumor macrophages are composed of several distinct populations that share features of both M1 and M2 macrophages. Thus, merely classifying tumor macrophages as M1 or M2 does not accurately reflect the differentiated or biological state of TAMs. Rather, the classification of TAMs should be related to the function of the macrophage subpopulation within the tumor (e.g., metastasis-promoting macrophage, angiogenic macrophage, and immunosuppressive macrophage) as has been proposed by others [44, 50, 53, 54]. For such classification purposes, researchers have relied primarily on the analysis of cell surface markers, none of which are entirely restricted to a specific subpopulation or lineage. In the murine setting, the absence of Gr1 (Ly6G) and the expression of the canonical markers CD11b, F4/80, and CSF-1R in combination with mRNA analysis of additional markers (Figure 3) are routinely used to classify macrophage subtypes [44]. In the human setting, antibodies to the glycoprotein CD68, the LPS-coreceptor CD14, CD312, CD115, HLA-DR, or Fc*γ*RIII (CD16) have been used to identify macrophages, but with mixed and oftentimes contradictory results [46]. Combinations of these markers provide higher specificity and should be used when possible to discriminate macrophages from other myeloid-derived cells, such as polymorphonuclear neutrophils and eosinophils. To more specifically identify M2-like TAMs and subsets, the hemoglobin-scavenger receptor CD163 [55, 56], the macrophage scavenger receptor 1 CD204 [53, 57, 58], the mannose receptor CD206 [59], and more recently the T-cell immunoglobulin and mucin-domain containing protein-3 (Tim-3) [60] have been used with great success. Ultimately, however, there remains considerable controversy regarding how to properly classify and identify TAMs. While classifications based on TAM functions, such as the promotion of angiogenesis or immunosuppression, are now being used to better categorize TAMs (Figure 3), it is important to note that macrophages are dynamic, plastic cells capable of performing many functions simultaneously. Thus, this approach may be self-limiting and underscore the multifunctional capabilities of TAMs. Since the scientific community has yet to come to a consensus regarding what markers to use and how to refer to macrophages, the binary M1/M2 classification remains commonly used [47].

TAMs directly participate in tumor initiation, progression, and metastasis via numerous mechanisms including (1) the secretion of proteolytic molecules such as MMPs to facilitate ECM remodeling [61–64], (2) the expression of nonproteolytic proteins like chemokines [65, 66], TGF-*β*1 [67, 68], and hCAP/LL-37 [69, 70] to facilitate tumor cell proliferation, migration, and invasiveness, (3) the expression of angiogenic mediators such as TGF-*β*, VEGF-A, VEGF-C, platelet-derived growth factor (PDGF), and MMP-9 to sustain the growth of the tumor stroma and promote* de novo* tumor blood vessel formation [44, 65, 71, 72], or (4) the expression of immunosuppressive factors including TGF-*β*, inducible nitric oxide synthase (iNOS), arginase-1, IDO (indoleamine 2,3-dioxygenase), and IL-10 to facilitate T-cell proliferation and activity [73–75]. While the mechanisms underlying the protumor effects of TAMs on bulk tumors have been extensively studied, there is now growing clinical and experimental evidence to support that TAMs also enhance tumor progression by directly communicating with CSCs to promote their stemness and/or subsequent oncogenic properties [76].

## 3. The Premalignant Niche

Normal adult stem cells occupy protective niches in various tissues where they function in tissue homeostasis and repair. The activity of stem cells in their tissue-specific niche is regulated by their own intrinsic molecular activity and the signals that they receive from neighboring differentiated cells [77, 78]. Increasing evidence, discussed below, suggests that macrophages interact with stem cells within their tissue-specific niche to modulate self-renewal and tissue remodeling in both normal and preinvasive tissues.

Alterations in tissue organization and homeostasis can precede tumor initiation, as exemplified by the increased cancer risk associated with chronic inflammation and wound healing. Moreover, epidemiological studies have shown that the administration of nonsteroidal anti-inflammatory drugs (NSAIDs) in low doses results in a significant decreased risk of developing colon, breast, esophageal, Hodgkin's lymphoma, pancreatic, and stomach cancer [79]. Thus, even before cancer begins, chronic inflammation or prolonged inflammatory episodes can set the stage for oncogenesis. The transcription factor nuclear factor-kappa B (NF*κ*B) is at the heart of cancer-related inflammation. In inflammatory cells, the NF*κ*B pathway results in the induction of numerous tumor-promoting chemokines and cytokines such as IL-6, TNF-*α*, IL-1*β*, CXCL8, VEGF, and CSF-1 [80]. In a mouse model of colitis-associated cancer, suppression of NF*κ*B in myeloid cells was shown to significantly decrease the incidence and size of tumors [81]. Subsequent studies showed that activation of NF*κ*B in macrophages leads to production of IL-6 and signal transducer and activator of transcription 3 (STAT3) signaling in neighboring cells, which promotes premalignant intestinal epithelial cell survival and CSC proliferation* in vivo* [82–84]. CCAAT/enhancer binding protein beta (C/EBP*β*) transcriptionally activates IL-6 in epithelial cells and is a direct target of IL-6 in macrophages. Interestingly, C/EBP*β* was shown to regulate stem cell self-renewal and maintenance in the normal mouse mammary gland [85], and C/EBP*β*, IL-6, and STAT3 are all overexpressed in preinvasive mammary hyperplasia as compared to normal mammary gland (H. Machado, unpublished data).

Interestingly, the effect of NF*κ*B activation on tumor initiation seems to be cell type-specific. In a diethylnitrosamine- (DEN-) induced model of HCC, mice with I*κ*B kinase beta- (IKK*β*-) deficient hepatocytes alone showed a significant increase in tumor number and size, which were characterized by increased reactive oxygen species (ROS), JNK signaling, and hepatocyte death. This cell death stimulated myeloid cells to produce mitogens such as IL-6, TNF-*α*, and HGF, which stimulated proliferation of the surviving hepatocytes. This effect was mitigated either when an antioxidant was administered to these mice or by conditional deletion of IKK*β* in hepatocytes and Kupffer cells [86]. While the role of CSCs in this model is unknown, studies using the normal mammary epithelial cell line, MCF10A, showed that activation of NF*κ*B leads to Lin28-mediated repression of Let7, resulting in a biphasic increase in IL-6 and ultimately self-renewal of CSCs [87]. NF*κ*B activation in infiltrating macrophages has also been tightly linked to pancreatitis and the development of pancreatic intraepithelial neoplasia (PanIN). During pancreatitis, acinar cells can undergo a transdifferentiation process known as acinar-to-ductal metaplasia (ADM) where their phenotype changes to a duct-like progenitor cell [88]. This process is driven by NF*κ*B-stimulated macrophage secretion of TNF-*α*, CCL5, and MMP-9 [89]. Once these duct-like progenitors are formed they can progress to PanINs if an oncogenic mutation is acquired, such as in KRAS [90]. Interestingly, a recent study showed that oncogenic KRAS signaling induces intracellular adhesion molecule-1 (ICAM-1) expression and the attraction of M1 polarized macrophages. Once recruited, these M1 macrophages promote ADM by secreting TNF-*α* and MMP-9 [91]. While M1 macrophages are generally believed to be “antitumor,” they may also contribute to oncogenic mutations by releasing reactive nitrogen and oxygen intermediates in premalignancy.

During inflammation, macrophages and other infiltrating leukocytes generate high levels of ROS and nitric oxide intermediates that generate DNA damage and genetic instability in epithelial cells. In addition, inflammatory cytokines and ROS deregulate DNA repair enzymes and p53 transcriptional activity leading to microsatellite and chromosome instability [83]. In mouse models with high levels of ROS, hematopoietic stem cells and oligodendrocyte/type 2 astrocyte progenitor cells have dramatically reduced self-renewal capacity due to the expression of senescence related proteins p16^INK4a^ and p19^Arf^ [92]. In tumors, CSCs upregulate cellular antioxidants to quench ROS [93, 94]. While the effect of ROS on CSCs in the preinvasive niche is not known, ROS scavenger proteins in CSCs may help select for their survival in premalignant lesions.

## 4. Primary Tumors

While TAMs in the preinvasive niche contribute to oncogenic transformation and survival, a growing body of evidence suggests that they are critical for the self-renewal and maintenance of CSCs in established tumors. STAT3 and NF*κ*B are key regulators of these processes. Once infiltrated into tumors, TAMs contribute to chronic inflammation by secreting inflammatory cytokines, such as IL-1*β*, IL-6, and IL-8 (CXCL8) [66, 95–97]. In breast cancer xenografts, IL-6 activates STAT3 by binding to its receptor (gp130) and directly stimulates breast CSC self-renewal [87]. Similarly, binding of IL-8 to the receptor CXCR1 promotes breast CSC expansion and prevents apoptosis [98]. Both of these cytokines are activated by the NF*κ*B pathway and, in a positive feedback loop mechanism, maintain and activate NF*κ*B [99]. In HCC, TAMs promote the expansion of CD44^+^ stemlike HCC cells in an* in vitro* coculture system. Furthermore, TAM-derived IL-6 induced CD44^+^ stemlike cell expansion by activating STAT3, and blocking IL-6 with tocilizumab ablated CD44^+^ sphere formation* in vitro* and tumor growth in patient-derived HCC xenografts [100]. Mitchem et al. showed that ablation of CCR2 or CSF-1R signaling significantly blocked TAM infiltration into pancreatic ductal adenocarcinoma (PDAC), decreased the number of CD44^+^ALDH1^+^ CSCs, and improved response to chemotherapy. Infiltrating TAMs also enhanced tumor-initiating properties of CD44^+^ALDH1^+^ pancreatic CSCs by activating STAT3 signaling [101].

IL-17 is another proinflammatory cytokine produced by macrophages and T-cells and has been shown to contribute to cancer-associated inflammation in numerous cancers [102–105]. Xiang et al. demonstrated that IL-17 promotes the self-renewal of ovarian CD133^+^ cancer stemlike cells through a mechanism involving NF*κ*B and p38 MAPK [106]. Using several different ER^+^ breast cancer cell lines, Ward et al. showed that coculture of M2 macrophages, but not M1 macrophages, increased tumor sphere formation* in vitro*, although the mechanism by which these macrophages promoted CSC expansion was not tested. Treatment of CSC spheres with zoledronate, a bisphosphonate currently used to treat osteoporosis and bone metastasis, reduced M2 macrophage-mediated sphere formation and migration [107].

The Sox family of transcription factors has also been shown to regulate CSCs in breast cancer. It is well known that a positive feedback loop exists between TAMs and tumor cells, involving epidermal growth factor (EGF) and CSF-1 [108]. Tumor cells secrete CSF-1 that promotes TAM production of EGF, and TAM-derived EGF stimulates tumor cell CSF-1 secretion. In mouse mammary tumor models, TAMs upregulate Sox 2 expression, which increases numerous stem cell genes including Sox-2, Oct-4, Nanog, and Sca-1. Inhibition of the EGF receptor (EGFR1) or STAT3 activation reduced Sox2 expression and CSC-associated phenotypes, suggesting a unique paracrine signaling pathway between TAMs and CSCs [109]. Overexpression of Sox-2 was also shown to increase breast CSC self-renewal by increasing tumor sphere-forming ability* in vitro* [110]. Sox-4, another pluripotency-associated gene, induced Ezh2 expression [111], which promoted breast CSC expansion by activating Raf-1 and *β*-catenin [112].

In addition to mediating CSC self-renewal and expansion, TAMs have been shown to be responsible for the maintenance of the CSC niche. A recent study by Lu and colleagues demonstrated juxtacrine signaling by TAMs and tumor-associated monocytes with mouse mammary CSCs to support the maintenance of a stemlike state [113]. EphH4 binding to its receptor on tumor cells resulted in the activation of Src and NF*κ*B, the latter of which caused the secretion of numerous cytokines that function in CSC maintenance. The IL-6/STAT3 pathway was also shown to increase tumor-initiating activities in murine colon and lung cancer cell lines by milk fat globulin epidermal growth factor-8 (MFGE-8). TAMs produced large amounts of both MFGE-8 and IL-6, which coordinately induced tumor potential and CSC chemoresistance through STAT3 and Hedgehog signaling, the latter of which regulates normal stem cell self-renewal. Interestingly, the MFGE-8 receptor, *α*_v_-integrin, was expressed in much higher levels on CSCs as compared to non-CSCs, further supporting a role for MFGE-8 in CSC maintenance [114].

While numerous studies have demonstrated that TAMs directly regulate CSC self-renewal and maintenance, there is a growing body of research that suggests that, in turn, CSCs recruit macrophages to solid tumors and enhance a protumor phenotype in TAMs. Zhou et al. recently showed that the extracellular matrix protein periostin is preferentially expressed on CD133^+^CD15^+^ glioma stem cells and recruits macrophages through integrin *α*_v_ *β*_3_ from the peripheral blood to the brain. Deletion of periostin in glioma stem cells resulted in decreased M2 TAM density, reduced tumor growth, and consequently increased survival in glioblastoma xenografts [115]. In pancreatic cancer, primary human PDAC CSCs (spheres) produce IFN*β*, which then induces the secretion of IFN-stimulated gene 15 (ISG15) in recruited TAMs. Consequently, TAM-derived ISG15 induced CSC self-renewal and tumor-initiating properties [116]. More recently, Sainz Jr. et al. demonstrated that PDAC CSCs secrete the TGF-*β* superfamily members Nodal/Activin A and TGF-*β*1, which then induce an M2 macrophage phenotype. Coordinately, polarized TAMs secrete the antimicrobial peptide hCAP-18/LL-37, which consequently binds to its receptors (formyl peptide receptor 2 (FPR2) and P2X purinoceptor 7 receptor (P2X7R)) to enhance CSC self-renewal, invasion, and tumor-initiating properties [70]. Of note, pancreatic CSCs also overexpressed two LL-37 receptors, further indicating a role for LL-37 in pancreatic CSC maintenance. In a different study, it was shown that PDAC CSCs induce an immunosuppressive phenotype in TAMs through STAT3, ultimately leading to chemoresistance [101]. Notably, the MFGE-8 receptor, which was shown to be preferentially expressed on CSCs in colon and lung cancer cell lines, can induce M2 polarization of macrophages* in vitro* though STAT3 signaling [117]. In summary, there exists a complex relationship between CSCs and TAMs in established tumors. It appears that macrophages are not just accidental passersby that happen to secrete CSC-promoting factors, but rather, CSCs attract, reeducate, and put macrophages into their service to support primary tumor growth. While researchers are just beginning to unravel the intricacies of these processes, there is no doubt that CSC-TAM cross talk represents an important component of CSC-mediated oncogenesis.

## 5. Circulating Cancer Stem Cells

Distant metastases have become the leading cause of death in patients diagnosed with cancer. Metastatic spread begins with cancer cells [known as circulating tumor cells (CTCs)] detaching from the primary tumor and entering into circulation, via either blood vessels or lymphatic channels in order to colonize distant sites. These cells must acquire the ability to overcome the challenges of the hostile extratumoral conditions and adapt to different tissue environments in secondary distant organs, such as the lungs, bone marrow, or liver. It is now commonly accepted that TAMs facilitate almost every step of the metastatic cascade, from initial migration to intravasation, dissemination, extravasation, and establishment of metastasis at secondary sites [44, 51]. One of the first definitive studies to highlight the role of TAMs in tumor metastasis was shown by Lin and colleagues in 2001. They demonstrated that CTC levels and lung metastases were significantly decreased in CSF-1-deficient mice as compared to wild type mice, supporting a role for tumor infiltrating macrophages in metastasis [118]. Additional studies targeting macrophages with clodronate liposomes, for example, have shown that elimination of macrophages significantly impacts CTC numbers and tumor metastasis [119, 120].

Once free from the tumor, CTCs can disseminate to distant organs to produce secondary metastatic lesions. Interestingly, only a minority of CTCs exhibit the capacity to successfully disseminate and proliferate in different organs, suggesting an internal hierarchy within CTCs. In fact, the existence of a small subset of CTCs with CSC properties has been shown for metastatic breast cancer [121], prostate cancer [122], small cell lung cancer [123], and PDAC [5], supporting the idea of a CSC compartment within CTCs that are distinct from CSCs of the primary tumor, enabling their escape to distant organs and subsequent growth. If this hierarchy within CTCs holds true, then TAMs likely facilitate the emergence of circulating CSCs and their intravasation and subsequent dissemination. The question remains, how do TAMs facilitate these processes in CSCs? While more studies are needed, a number of experimental systems are beginning to provide evidence that TAMs can promote an epithelial-to-mesenchymal transition (EMT) phenotype in CSCs via paracrine-secreted factors. Loss of epithelial differentiation, the acquisition of a migratory phenotype, and loss of cell adhesion are hallmarks of EMT. This process is regulated by numerous genetic modifications and a panel of well characterized transcription factors, such as SNAIL, TWIST, ZEB1, ZEB2, SLUG, BMI-1, and LOXL2 [35, 124, 125]. While numerous studies have shown that TAMs can promote an EMT phenotype in non-CSCs [68, 126–131], TAM-mediated EMT induction in CSCs was largely unappreciated until recently. In the context of pancreatic cancer, two recent studies showed that CSCs isolated from patient-derived PDAC xenografts and treated with conditioned media from M2-polarized monocyte-derived macrophages increased migration and expression of EMT genes [70, 116]. The authors identified the human cathelicidin antimicrobial peptide LL-37 and ISG15 as independent TAM-secreted mediators of these phenotypes in pancreatic CSCs. Similar TAM-mediated EMT induction has been observed in CSCs of HCC [126] and ovarian cancer [69]. STAT3 activation of target genes such as TGF-*β*1 and hypoxia inducible factor- (HIF-) 1*α* has been linked to EMT reprograming [132] and several recent studies have shown that TAM-secreted IL-6, EGF, or MFGE-8 can activate STAT3 signaling in CSCs of breast cancer [109, 133], HCC [100], or colon cancer [134]. Thus, apart from activating these pathways in CSCs to promote tumor growth as discussed above, TAM-mediated STAT3 activation may also be necessary for EMT reprogramming in CSCs. While the aforementioned studies highlight that EMT and “stemness” may go hand in hand, the implications reach beyond merely the induction of a migratory and invasive phenotype. For example, EMT transactivators have been associated with the maintenance of stem cell properties and cell survival [135], and more recently EMT induction has been shown to produce* de novo* breast CSCs [135] and to facilitate CSC maintenance in pancreatic cancer [136]. Thus, while the TAM-mediated induction of EMT in CSCs is likely necessary for the generation of migratory CSCs with invasive capacities, the implications of an EMT transcriptional signature in CSCs may be more dynamic than previously thought.

In addition to paracrine-mediated signaling, juxtacrine signaling from macrophages represents an alternate means by which TAMs can communicate with CSCs. Intravital imaging revealed that tumor cells and macrophages interact in a contact-dependent manner and comigrate* in vivo*, tumor cell migration is dependent on juxtacrine signaling, and the efficient long-distance comigration and eventual intravasation of these cells are coordinated by an EGF-CSF-1 paracrine loop [reviewed in [137]]. Along these lines, Lu et al. recently showed that TAMs physically interact with mouse breast CSCs via CD11b binding to the CSC marker CD90, leading to ephrin ligand binding to EphA4, the activation of Src and NF*κ*B, and the subsequent secretion of various cytokines that, in turn, function to maintain the stemlike state of CSCs [113]. Taken together, these cell-cell contact-dependent interactions provide evidence of a physical CSC niche supported by TAMs; however, it is also plausible that, apart from merely interacting, CSCs and TAMs may actually fuse with one another to create a macrophage-tumor circulating cell with recombination/reprogramming of genetic material [138], analogous to that observed in stem cell fusions studies [139]. This concept, loosely known as epithelial-myeloid transition [140], was first proposed by the German pathologist Otto Aichel in 1911 to explain how a cancer cell could efficiently travel through the circulatory and lymphatic systems, while maintaining their cancer cell growth properties. Since then, the concept has slowly gained momentum [141, 142]. However, with the recent discoveries of CTCs expressing both cancer and leukocyte cell markers [143–145], the idea of “mobile hybrids” resulting from fusion events between TAMs and tumor cells is evolving as a more tangible explanation behind metastasis. Regardless of how TAMs promote CSC invasion, as stated by Qian and Pollard, macrophages “are the key that unlock the gate to allow tumor cells to escape” [44].

## 6. Premetastatic Niche

While many tumor cells have a predilection for metastasis, only a small percentage of CTCs (less than 0.2%) have the capacity to survive in circulation, find a suitable secondary site to support their colonization, and proliferate in their new environment [146]. In fact, apoptosis of tumor cells entering target organs represents a common early event during metastasis [147, 148], severely limiting the colonization efficiency of CTCs. Thus, while successful intravasation initiates the metastatic process, efficient survival and proliferation determine the outcome. The “seed” and “soil” theory put forth by Paget in 1889 suggested that the secondary organs themselves provide the appropriate conditions (i.e., “soil”) necessary for metastatic colonization by CTCs. Our current take on Paget's theory now combines “organ selectivity” with “cell fitness,” meaning that CTCs must also be genetically (i.e., accumulate specific mutations) or epigenetically programmed for metastasis. CSCs inherently possess the necessary “fitness” and programs for dissemination, and at the same time they bear the functional plasticity needed for transitioning between mesenchymal-like and epithelial-like states [149], the latter being necessary for CSCs to seed and resume growth at the metastatic site. In 2006, Balic et al. first linked metastasis to CSCs by demonstrating that disseminated breast cancer cells in bone marrow possessed stem cell phenotypes [150]. One year later, Hermann et al. showed that tumor metastasis in PDAC is driven by a distinct subpopulation of CD133^+^ CXCR4^+^ CSCs in the invasive front [5]. Today, CTCs have been shown to coexpress EMT and multiple stem markers, suggesting that CSCs are present within the CTC population [151].

In light of ever growing data supporting a role for CSCs as the “seed,” CSCs are also susceptible to the harsh conditions faced during dissemination and not all cells bearing CSC markers are metastatic. Thus the “soil” counterpart of Paget's theory must also be important for CSC-mediated metastasis. Indeed, it has become evident that the formation of CSC-promoting premetastatic niches in secondary organs is not only essential but also necessary for successful CSC colonization, and current evidence suggests that resident or infiltrating immune cells, specifically macrophages, at distant sites drive the creation of premetastatic niches to facilitate successful establishment of secondary lesions. One of the earliest studies to support this hypothesis showed that not only do macrophages facilitate the growth of extravasated tumor cells, but also their elimination after initial cancer cell dissemination had been established led to a significant decrease in lung metastasis. Thus, the presence of macrophages in secondary organs is necessary for successful CTC extravasation, establishment, and growth [152].

Whether TAMs are present before the arrival of circulating CSCs or whether they are recruited following CSC extravasation remains unclear. In mouse lung or melanoma subcutaneous tumors, CD11b^+^ myeloid cells accumulate in the lungs prior to the detection of metastatic tumor cells [153]. In studies using a genetically engineered mouse model of PDAC, infiltration of F4/80^+^CD11b^+^ macrophages in the livers of mice was observed months before tumor development and metastatic growth (M. Vallespinós and B. Sainz Jr., unpublished data). There is increasing evidence that more differentiated myeloid cells also play an important role in the development of the premetastatic niche. Specifically, van Deventer et al. observed that the recruitment of CD11b^+^Ly6C^+^ monocytes to the premetastatic lung enhances B16 cell metastasis [154], and Gil-Bernabé et al. demonstrated that CD11b^+^CD68^+^F4/80^+^ recruited macrophages establish the premetastatic niche that facilitates successful breast cancer metastasis to the lungs [155]. It remains to be determined if the sum of these findings holds up in the human setting. Until then, it is interesting to speculate that primary tumor-derived secreted factors, such as soluble proteins or exosomes [156], precondition the premetastatic sites in different organs by preloading them with recruited myeloid progenitor cells. Once recruited to these sites, they can rapidly differentiate into metastasis-associated macrophages (MAMs) following the arrival of circulating CSCs, thus facilitating CSCs extravasation, survival, and subsequent proliferation via paracrine-mediated mechanism [157]. It is also important to note that, like TAMs in the primary tumor, MAMs may also facilitate CSC survival from immune cell destruction via the immunosuppressive mechanisms discussed above. Thus, the contribution of macrophages in the premetastatic and their influence in the development of metastatic lesions may be more important than their role in the primary tumor.

## 7. Therapeutic Strategies

Cancer has been treated with radiation therapy, chemotherapeutic drugs, and hormonal therapy for decades; however, these treatments are not tumor cell-specific and can result in severe toxicity. Tumor cells have acquired the ability to circumvent the effects of conventional therapies, leading to resistance to anticancer therapies. While there has been a recent explosion in the field of developing targeted molecular therapies that specifically block tumor cell growth and progression, a subset of cells can evade the effects of these drugs, leading to drug-resistance and/or tumor relapse. The question remains as to whether we are targeting the right population of cells.

Numerous antimacrophage strategies, including trabectedin [158], RG7155 (anti-CSF-1R) [159], and an anti-MIF (macrophage migration inhibitory factor) antibody [160], have been developed and are currently being tested in preclinical and Phase I clinical trials. However, the CSC model suggests that effective therapeutic strategies must target CSCs to not only eliminate tumor progression, but also prevent tumor recurrence after therapy. As the tumor microenvironment provides CSCs with protection from conventional therapies by promoting their “stemness” and CSCs enhance protumor properties of TAMs, disrupting CSC-TAM cross talk, or using a combined strategy to target both CSCs and TAMs, represents an exciting and promising approach for cancer therapy. A recent study demonstrated that cancer stemlike cells from chemoresistant tumors release proinflammatory cytokines that contribute to a protumor microenvironment by generating M2-like myeloid cells [161]. Mitchem and colleagues showed that targeting TAMs in PDAC reduced both CSC properties and chemoresistance [101]. These results suggest that targeting the CSC-TAM interaction is crucial for not only preventing tumor progression, but also circumventing chemoresistance.

One of the most promising antibody-mediated therapeutic strategies to date is based on inhibiting the interaction between SIRP*α* and CD47, a transmembrane protein expressed on many cancer cells and CSCs [162, 163], to allow for increased phagocytosis of cancer cells. Interaction of CD47 (“don't eat me” signal) with SIRP*α* results in the inhibition of phagocytosis by macrophages (including TAMs) through a signaling cascade mediated by phosphorylation of the immunoreceptor tyrosine-based inhibitory motif present on the cytoplasmic tail of SIRP*α* [164]. Numerous studies over the past few years, predominantly led by Weissman and colleagues, showed that blocking CD47 using anti-CD47 monoclonal antibodies allows for increased phagocytosis of cancer cells* in vitro* and decreased tumor burden* in vivo* [162, 163, 165, 166]. Recent work by Cioffi et al. has extended these findings to show that anti-CD47 therapy can essentially turn the tide on the relationship between CSCs and TAMs, facilitating effective phagocytosis of pancreatic CSCs, which can be further augmented with standard chemotherapeutic agents like gemcitabine or Abraxane [162]. These findings suggest that CD47 inhibition in the adjuvant setting may be an effective means for treating PDAC and potentially other cancers; however future preclinical and clinical studies will need to be performed. As we gain a better understanding of the relationship between TAM and CSCs at each stage of tumor development and progression, we will undoubtedly discover new means to interfere with the TAM-CSC cross talk.

## 8. Concluding Remarks

**Figure 4:**
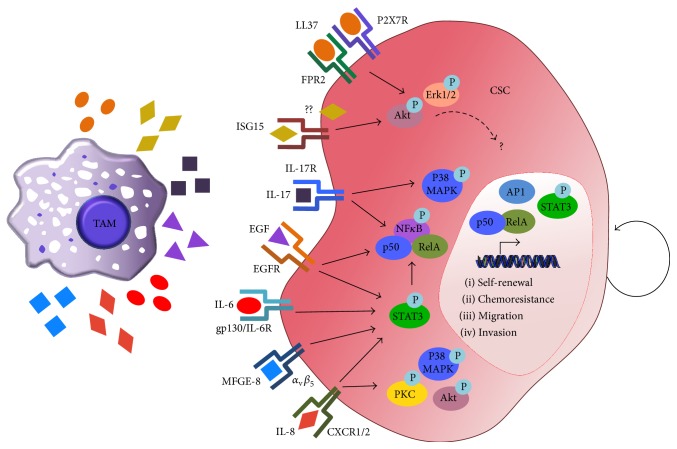
TAM-secreted factors regulate CSC phenotypes. TAMs have been shown to secrete LL-37, ISG15, IL-17, EGF, IL-6, MFGE-8, and IL-8 (among others), which in turn activate MAPK, STAT3/NF*κ*B, and other yet-to-be-defined signaling pathways, leading to the activation of CSC properties, such as self-renewal, chemoresistance, migration, and invasion.

In this review, we discussed several TAM-derived factors that promote stemness and are thus potential therapeutic targets (summarized in Figure 4). The studies of the past decade have led to significant advances of our understanding of the molecular pathways regulating TAMs and CSCs; however, we are only beginning to put together the pieces that constitute the complex TAM-CSC cross talk that occurs within the host. Increasing evidence suggests that a stemlike niche composed of numerous cell types, including macrophages, is important for promoting CSC self-renewal and maintenance, and likewise, CSC-derived factors induce protumor signals in TAMs. Our current knowledge of CSCs heavily relies on tumor transplantation assays in both syngeneic and xenograft models, the latter of which does not recapitulate the complex microenvironment in which spontaneous tumor initiation occurs, nor can xenograft models accurately mimic human CSC and human TAM interactions. While many immune-compromised mice express macrophages, the macrophage response is typically elevated in these mice and it is uncertain as to whether murine macrophages communicate with human CSCs in the same way as their human counterparts. Thus, until we develop mouse models with humanized immune systems that can support the growth of human primary tumors, we will continue to rely on excellent* in vitro* systems and syngeneic mouse models to better facilitate our understanding of the relationship between TAMs and CSCs and the eventual development of novel compounds to inhibit this unconventional dependence.
